# How the Stakeholders’ Perception Contributes to the Pharmaceutical Strategies: A Regional Case Study in Latin America

**DOI:** 10.3390/jmahp13040054

**Published:** 2025-10-23

**Authors:** Talita da Silva Ferreira, Giovanni M. Pauletti, Luis Vázquez-Suárez

**Affiliations:** 1 Department of Business Administration, Instituto Multidisciplinar de Empresa (IME), Campus Unamuno, Edificio FES, University of Salamanca, 37007 Salamanca, Spain; lvazquez@usal.es; 2 Department of Pharmaceutical and Administrative Sciences, University of Health Sciences and Pharmacy in St. Louis, 1 Pharmacy Place, St. Louis, MO 63110, USA; giovanni.pauletti@uhsp.edu

**Keywords:** collaboration, strategies, pharmaceutical, stakeholders

## Abstract

**Background**: Stakeholders’ perception plays a crucial role in shaping pharmaceutical strategies. Stakeholders are groups interested in pharmaceutical companies’ success and outcomes. Stakeholders’ perceptions are multifaceted and impact pharmaceutical strategies, from shaping research to enhancing market access, pricing, and corporate reputation. Understanding and actively managing stakeholders’ perceptions is vital for pharmaceutical companies to succeed in an increasingly complex and competitive industry. **Methods**: In this case study, knowledge contributions from stakeholders offered insights and strategies for application in the pharmaceutical sector. **Results**: Qualitative, exploratory research was conducted, which included the participation of sixteen stakeholders from different countries in Latin America, who responded to a semi-structured interview script, whose data were understood through lexical analysis in the Interface de R pour les Analyses Multimensionnelles de Texts et de Questionnaires (IRaMuTeQ). **Conclusions**: The results of this study underscore the importance of regulatory knowledge for professionals’ support and implementation of international strategies. Regulatory knowledge provides professionals with tools and insights to navigate complex regulatory environments, make informed decisions, and enhance organizational performance in global markets.

## 1. Introduction

### 1.1. Regulatory Knowledge

The definition of organizational learning [1] highlights the importance of knowledge acquisition and its application to enhance an industry’s performance. In the pharmaceutical sector, regulatory knowledge is a critical subset of the knowledge required for successful drug development and market authorization. Organizational learning plays a crucial role in competitive advantage in the pharmaceutical industry and is achieved through effective acquisition, sharing, and utilization of regulatory knowledge [2].

Regulatory knowledge comprises both tacit and explicit dimensions. Tacit knowledge is informal, experiential, and practice-based, developed through the daily activities of regulatory professionals. Because it is embedded in individual experience, it is often difficult to articulate, codify, or transfer. It includes insights from hands-on experience, navigating the complexities of regulatory processes in different countries, understanding regulatory authorities’ preferences, and effective communication during submissions and reviews.

On the other hand, explicit knowledge in the regulatory context represents the formal, codified information that can be documented, shared, and communicated efficiently [3]. It includes regulatory guidelines, documentation requirements, submission processes, and regulatory databases.

The combination of tacit and explicit knowledge is vital for regulatory professionals when applying for market authorization [4]. Leveraging both forms of knowledge facilitates the development of effective strategies, streamlines the regulatory approval process, and ensures compliance with local regulations, ultimately leading to successful market entry for new pharmaceutical products.

Understanding the dynamics of regulatory knowledge, how it is acquired, shared, and utilized within pharmaceutical organizations, and its impact on market authorization processes can provide valuable insights for enhancing regulatory strategies, optimizing drug development, and ensuring global compliance in the pharmaceutical sector. As the industry evolves, studying regulatory knowledge in detail improves regulatory affairs and decision-making in the pharmaceutical sector.

While research on tacit and explicit knowledge is well-established, further studies and empirical research are needed to delve deeper into regulatory expertise within the pharmaceutical context. Thus, this study analyzed the critical aspects of knowledge related to international strategies in the pharmaceutical sector, emphasizing that understanding and actively managing stakeholders’ perceptions is essential for pharmaceutical companies to succeed in an increasingly complex and competitive industry.

### 1.2. Knowledge-Based Approach

Knowledge is widely regarded as a company’s most critical strategic resource [1]. To remain competitive in specialized markets, firms must develop the ability to effectively manage and leverage this resource. As Grant [1] emphasizes, the firm’s primary role is to integrate and apply both tacit and explicit knowledge possessed by its professionals in order to formulate and implement competitive strategies.

The interviews were conducted with representatives from health authorities and industry specialists, selected based on their direct experience with internationalization processes and regulatory knowledge. Stakeholders from 16 Latin American countries participated in the study, ensuring geographic and institutional diversity. The interviews took place remotely between 2021 and 2022, following a semi-structured script that allowed for the exploration of technical, strategic, and contextual aspects related to regulatory practices in the region.

Nonaka et al. [3] highlight that tacit knowledge originates from within individuals and is unique to each professional. Because it is highly personal and experience-based, it is difficult to capture, store, or formalize, relying instead on interpersonal interaction for transfer. By contrast, explicit knowledge—codified in books, articles, and documents—can be systematically collected from both internal and external sources and more easily disseminated across the organization. According to these authors, the dynamic interconnection between tacit and explicit knowledge is essential to foster continuous learning and innovation at both the individual and organizational levels.

Hitt et al. [5] argued that developing strategies to expand a company’s business to other markets is challenging. Companies must use appropriate resources to implement international strategies. They must diversify internationally for various reasons, including acquiring unique knowledge about foreign countries [5].

In the pharmaceutical context, regulatory knowledge integrates both tacit and explicit dimensions, forming a comprehensive body of data, information, and expertise that regulatory professionals must apply to obtain marketing authorization. The resulting documentation package constitutes the formal submission for Market Authorization (MA), which is subsequently evaluated by health authorities prior to the approval of a new drug product. Beyond its compliance function, regulatory knowledge can serve as a strategic asset, providing companies with a competitive advantage in designing and implementing internationalization strategies within the pharmaceutical industry.

Devarakonda and Reuer [6] examined the mechanisms of knowledge transfer between companies and universities in the development of pharmaceutical products. Their analysis highlights the importance of collaborative partnerships in combining scientific expertise with industrial capabilities to accelerate innovation and product development. Knowledge of pharmaceutical companies’ diversification processes was essential to implementing internationalization strategies in other countries. Building on this perspective, Lilleoere and Hansen [7] investigated the sharing of tacit and explicit knowledge within a European pharmaceutical company, focusing on R&D professionals from diverse cultural backgrounds. Their findings suggest that the integration of tacit and explicit knowledge not only reduces time to market but also creates a sustainable competitive advantage in the pharmaceutical sector. Although based on a single case study, the insights can be generalized and applied to other companies and international contexts, underscoring the strategic value of knowledge sharing in global pharmaceutical development.

Nonaka et al. [3] argue that firms must continuously explore and exploit knowledge to ensure competitiveness and sustainable growth. One key process in this regard is internalization, through which knowledge is absorbed and applied within the organization. In line with this perspective, Cherchem and Keen [8] examined the relationship between knowledge exploitation, internationalization strategies, and stakeholder perceptions. Their findings indicate that intellectual capital exerts a positive influence on the development of international strategies, with the notable exception of the disruptions caused by the COVID-19 crisis.

Therefore, pharmaceutical companies can make informed decisions about positioning their products, collaborate with local stakeholders, and navigate the complex web of international regulations and market dynamics. This holistic approach fosters a deeper understanding of the needs and expectations of diverse markets, more effective market entry, successful product launches, and improved healthcare outcomes for patients worldwide.

### 1.3. Regulatory Knowledge Diversity

Regulatory knowledge of pharmaceutical products is crucial for international markets. Each country has its own set of regulations and requirements for importing, manufacturing, distributing, and selling pharmaceutical products.

Each country’s approach to regulatory knowledge is shaped by its cultural, political, and economic context, as well as its regulatory environment. In Latin America, regulatory knowledge tends to be more informal compared to the United States and Europe, reflecting weaker legal systems and a more flexible approach to regulation. The region’s regulatory landscape is complex and challenging to navigate, given the differences across countries and the varying levels of regulatory enforcement. Nonetheless, there is a growing movement toward regional standardization and harmonization, particularly in the regulation of pharmaceuticals and medical devices, which aims to reduce fragmentation and facilitate market access.

The health authorities for regulation in Latin America are seen in Table 1.

**Figure 1:**
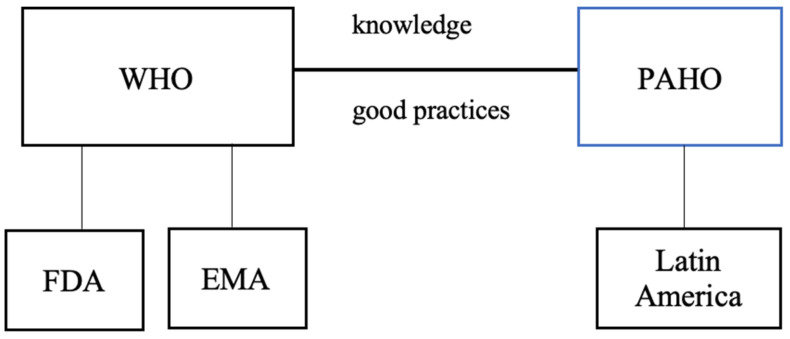
Regulatory Frameworks for FDA, EMA, and Latin America. Abbreviations: World Health Organization (WHO); U.S. Food and Drug Administration (FDA); European Medicines Agency (EMA). Source: Researcher (2022).

The World Health Organization (WHO) collaborates with regulatory authorities worldwide, including the U.S. Food and Drug Administration (FDA) and the European Medicines Agency (EMA). Although WHO works in close partnership with these agencies on global health issues, both the FDA and EMA operate independently, maintaining distinct regulatory frameworks and responsibilities within their respective jurisdictions. In the Americas, the Pan American Health Organization (PAHO) functions as the WHO regional office, supporting Latin American countries in strengthening health systems, addressing public health challenges, and promoting health and well-being (Figure 1).

PAHO/WHO actively promotes the exchange of good practices and cooperation among countries in the Americas to strengthen essential public health roles. By facilitating collaboration and technical assistance, PAHO/WHO supports countries in enhancing their public health systems and achieving better health outcomes for their populations.

The strategy to harmonize pharmaceutical regulations in the region relies on the active involvement of professionals who ensure that industry standards are properly defined and evaluated by regulatory authorities. In this context, the harmonization of technical and regulatory knowledge emerges as a key approach to fostering collaboration among health authorities, pharmaceutical companies, and academic institutions. Such efforts aim to align requirements for the introduction of generic medicines across countries, thereby reducing regulatory barriers and facilitating regional market integration.

### 1.4. International Strategies

Expanding into international markets is an excellent strategy to understand different markets, cultures, and business practices. The best approach is to seek collaborations or partnerships with local companies, distributors, or industry experts who deeply understand the target market. Their expertise can provide useful insights and guidance throughout market entry.

**Figure 2:**
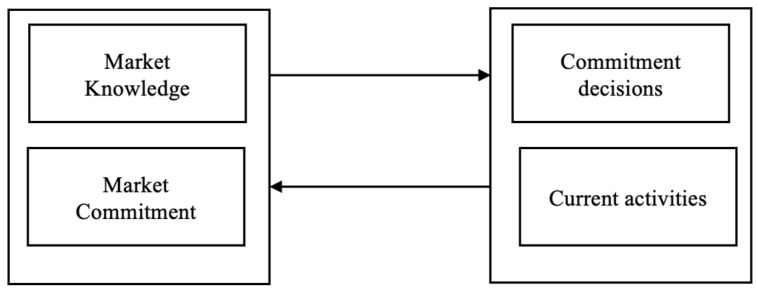
The basic mechanism of internationalization is called the Uppsala Model [9]. Source: Adapted from Johanson & Vahlne (1977) [9].

The assumption in the internationalization process model [9], often called the Uppsala model, involves accumulating experience about the domestic market before venturing into international markets. The assumption applies to companies of any size. The model provides insights into how companies gradually internationalize operations and increase their commitment to foreign markets over time. The model is based on two key concepts: the “knowledge-based view” and the “commitment-based view” (Figure 2).

The Knowledge-Based View suggests that companies accumulate experience through international activities. As companies engage in global operations, they gain insights into foreign markets, customer preferences, distribution channels, and business practices. Knowledge develops through learning about market research and interactions with local partners. The more knowledge a firm acquires, the more effectively it navigates and adapts to foreign markets.

The Commitment-Based View model emphasizes the gradual increase and commitment to foreign markets as the companies gain knowledge and experience. Commitment refers to allocating resources, both tangible (e.g., investments and assets) and intangible (e.g., managerial attention and relationships), to international activities. Initially, companies’ low commitment involves less risky market entry modes, such as exporting or licensing. However, as experience grows, companies become more involved and may establish subsidiaries or engage in joint ventures or acquisitions.

According to Zahra et al. [10], international market entry is accomplished through direct or indirect modes (exporting, licensing, alliances, and start-ups). Lin & Ho [11] understand that other modes exist, such as franchising, management contracts, turnkey contracts, subcontracting or associations, and consortiums. However, the modes mentioned by Zahra et al. [10] are the most common in the pharmaceutical sector.

International market entry faces challenges involving cultural differences, language barriers, legal protection, currency exchange, and political influences. Hitt et al. [5] emphasize that companies acquire unique knowledge when accessing foreign countries despite uncertainties. Aggarwal [12] analyzed how regulatory professionals conduct strategic alliances. He identified that biopharmaceutical companies use knowledge-based resources to partner with other companies for alliance portfolios. Although the study focused on biopharmaceutical R&D alliances, it analyzed companies located only in the United States.

Additionally, government-based committees can increase partners’ interaction in biotechnology alliances [6]. Dow et al. [13] explored direct and indirect international entry modes for developing a local subsidiary (licensing) and forming joint ventures (alliances) with regional players. Partnerships with local companies can gain fundamental knowledge of international market access.

According to Martín Martín et al. [14], international business is crucial for effective marketing strategies, tailoring products or services to meet local demand, and identifying competitive advantages in foreign markets. Companies adapt their business models and marketing approaches to reach the target market and gain a competitive edge. Finally, interaction with distinct stakeholders in foreign markets is a critical resource for effective communication [13].

## 2. Methods

Knowing each country’s regulatory requirements and health practices in the pharmaceutical sector is essential for entering new markets. Understanding specific regulations and healthcare systems for each country helps regulatory professionals develop appropriate strategies for introducing new products, complying with local laws, and building relationships with other stakeholders.

Exploratory research was carried out, with a qualitative approach, which seeks to delve deeper into this topic, little explored in the literature. Qualitative methods, such as interviews in case studies, could capture the nuanced and subjective experiences of participants, which are central to understanding the psychological dynamics at play. The participants answered the questions based on three thematic axes:

- Regulatory experience;
- Internacional modes;
- Internalization strategies.

In this context, the research aimed to gain deeper insights into personal, emotional, and cognitive experiences that aren’t easily captured through more quantitative and structured approaches. As Yin [15] suggests, focusing on the psychological experiences of participants is often vital to understanding intricate aspects of a topic.

### 2.1. Data Analysis

The interview data were analyzed using the software *Interface de R pour les Analyses Multidimensionnelles de Textes et de Questionnaires* (IRaMuTeQ), version 0.8 alpha 7. The analysis followed four main steps. Initially, classic lexicographic analyses were performed, with the aim of verifying statistics related to the number of evocations, forms and text segments (TSs)—excerpts of sentences with approximately three lines. Then, the Descending Hierarchical Classification (DHC) was applied, which made it possible to identify the dendrogram with the classes formed.

Based on the content distributed in each class generated by IRaMuTeQ, a content analysis was performed, and a Word Cloud was generated, which graphically organized the terms based on their relevance. In this case, the largest words represent the highest frequency, and only those with a minimum frequency of 10 were considered.

IRaMuTeQ was selected for its capacity to conduct multidimensional lexical analyses and to generate dendrograms that visually depict thematic clusters. In comparison with software such as NVivo and Atlas.ti, IRaMuTeQ provides a more automated and statistically grounded approach to textual data processing, aligning with this study’s objective of identifying dominant discursive patterns in stakeholder interviews. Moreover, its use enhanced the reproducibility and transparency of the analysis—two essential elements in qualitative research with an interpretive orientation.

### 2.2. Participants

The methodology used here to understand international market entry was a case study—the collected data involved interviews to understand the case comprehensively [15]. The case study was conducted in a real-world context in the pharmaceutical sector, involving 16 stakeholders from different health authorities in Latin America. The stakeholders that participated in this research were from Chile, Colombia, Brazil, Cuba, Argentina, Paraguay, El Salvador, Mexico, Panama, Peru, Bolivia, Costa Rica, Honduras, Nicaragua, Dominican Republic, and Uruguay.

Participants were selected through a purposive sampling strategy, given their expertise and direct involvement in internationalization processes within the pharmaceutical and healthcare regulatory sectors in Latin America. Additionally, a snowball approach was applied, as some stakeholders referred us to other relevant professionals with complementary experiences. Data collection continued until thematic saturation was achieved: after the 14th interview, no new categories or relevant insights emerged, and two additional interviews were conducted to confirm saturation, resulting in a total of 16 participants. While qualitative research does not aim for statistical representativeness, the diversity of participants—in terms of countries of origin, organizational roles, and experiences—ensured sufficient breadth and depth to capture varied perspectives and provide robust, credible findings aligned with the study objectives.

The inclusion criteria comprised stakeholders with regulatory experience in the internationalization of pharmaceutical products who were members of a health authority in Latin America. The exclusion criteria included stakeholders without direct regulatory responsibilities, professionals from outside the pharmaceutical sector, and individuals not affiliated with a Latin American health authority.

This study did not require submission to a Research Ethics Committee, as established by Brazilian regulatory requirements, since it did not involve patients, clinical data, or direct health interventions. The research was based exclusively on voluntary interviews with professionals regarding their experiences and perceptions. All participants were informed about the objectives of the study and voluntarily agreed to participate. To ensure confidentiality and compliance with ethical standards, personal identifiers (such as names, companies, locations, and gender) were anonymized, sensitive information was not disclosed, and all data were securely stored. The procedures adopted are in accordance with the Brazilian General Data Protection Law (Lei Geral de Proteção de Dados—LGPD) and international guidelines for ethical research involving human participants.

## 3. Results

Latin America has made progress in regulatory harmonization initiatives, particularly in the areas of pharmaceutical products and medical devices, through good regulatory practices and reliance mechanisms. However, the regulatory environment remains fragmented, with varying levels of maturity among countries, fragile legal systems, and flexible approaches that hinder process predictability. In contrast, the European Union, through the EMA and the new HTA regulation, operates with standardized guidelines, mandatory joint clinical assessments, and formal stakeholder participation, promoting transparency, consistency, and equitable market access. While Latin America continues to build its regulatory foundation with the support of informal networks and technical cooperation, the European model offers a consolidated structure that may serve as a strategic reference for the region.

The stakeholders who knew about international entry modes likely possessed a deeper understanding of the requirements and intricacies involved in expanding operations to foreign markets. They know regulatory compliance in different countries, identify market opportunities through analysis, establish effective distribution channels, form strategic alliances with local partners, and deploy global marketing strategies to promote their products or services internationally. The interviews discriminated the stakeholders well informed on developing targeted interventions to facilitate successful international market entry.

According to the stakeholders, the key differentiating factor is the level of regulatory knowledge and expertise possessed by companies. Larger and multinational pharmaceutical firms are more likely to succeed in implementing internationalization strategies, owing to their greater resources, accumulated experience, and established presence across multiple markets (Table 2). Table 2 provides a comprehensive compilation of the results obtained from the stakeholder interviews.

An in-depth analysis of the open-ended responses may provide richer insights that go beyond the quantitative findings. These narratives can reveal specific challenges faced during internationalization efforts, such as difficulties in meeting divergent regulatory requirements, adapting to new market entry strategies, or building trust with local stakeholders. At the same time, success stories highlight effective practices, including knowledge transfer, capacity building, and innovative approaches that facilitated smoother expansion processes.

Stakeholders’ perspectives are particularly valuable, as they shed light on the multifaceted nature of global expansion. They emphasize not only the technical aspects of complying with heterogeneous regulatory frameworks but also the human and organizational dimensions, such as managing cultural differences, overcoming language barriers, and aligning internal capabilities with external market expectations. Moreover, these accounts capture the operational nuances of navigating distinct legal systems, negotiating with local authorities, and tailoring products or services to different consumer behaviors. Altogether, the qualitative analysis complements the quantitative data and deepens the understanding of the complexities inherent in internationalization.

Stakeholders’ perspectives offer helpful information on the intricacies of global expansion, cultural differences, language barriers, and the details of operating in different regulatory environments.

These three strategies—harmonization of documents, partnerships, and regulatory compliance—reflect the multifaceted nature of international market entry in the pharmaceutical industry. Each strategy addresses different aspects of the process, from regulatory approvals to market access and collaboration with local stakeholders.

Understanding diverse points of view helps policymakers, industry players, and regulatory authorities understand the complexities of international market entry. It provides a basis for targeted support programs, capacity-building initiatives, and policies that facilitate and accelerate the globalization of the pharmaceutical industry. Insights from stakeholders emphasize the investment in regulatory knowledge, forming strategic partnerships, and ensuring compliance to enhance the success of internationalization strategies in the pharmaceutical industry.

### 3.1. Descending Hierarchical Classification

**Figure 3:**
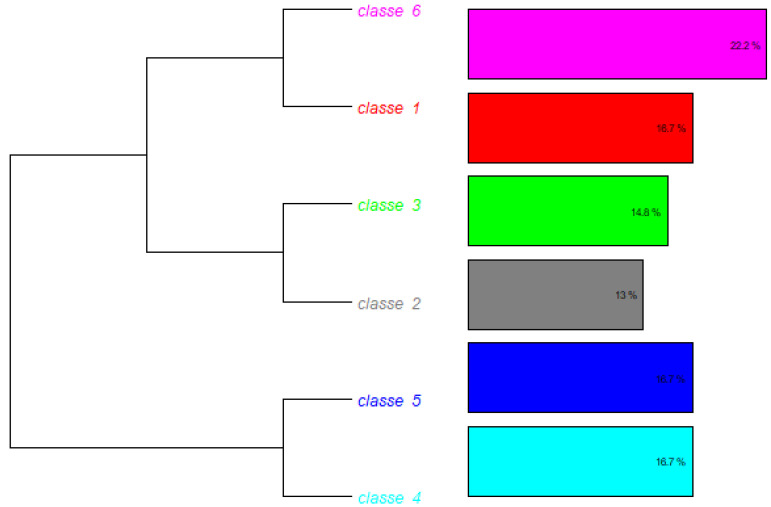
Descending hierarchical classification per class. Source: Researcher (2022).

The general corpus consisted of 66 text segments (TSs), of which 54 (81.82%) were retained for analysis. A total of 2,241 occurrences (words, forms, or vocabulary) were identified, comprising 510 distinct words, 442 of which appeared only once. The analyzed content was subsequently categorized into six classes (Figure 3).

Basic lexicographic statistical analysis:

Number of texts: 16

Number of text segments (TSs): 66

Number of forms (different words): 510

Number of occurrences (total words): 2241

Number of lemmas (reduced forms, such as word roots): 442

Number of active forms: 328

Number of supplementary forms: 114

Number of active forms with frequency ≥ 3: 106

Average number of forms per segment: 33.95

Number of classes identified: 6

Classified segments: 54 of 66 (81.82%)

- Class 6 represented the largest in percentage, indicating that it brings together the largest number of segments with common characteristics. The associated terms suggest themes such as economic conditions; regulatory approaches; international partnerships; and local investments.

This class represents 22.2% of the total corpus (f = 6 TS). It is characterized by words such as ‘*condition*’ (χ^2^ = 12.343), ‘*economic*’ (χ^2^ = 10.643), ‘*environment*’ (χ^2^ = 9.907), and ‘*partnership*’ (χ^2^ = 6.962).

The choice of an expansion mode is one of the most critical decisions a company makes when entering new markets. It affects the company’s level of control over its operations, the financial investment required, and the risks it faces in that market. Each mode of expansion comes with its own set of benefits and trade-offs, and companies need to carefully consider how these align with their business strategy, resources, and risk tolerance.

The participant understands that

“*These international modes refer to the various approaches companies can use to expand their operations into foreign markets. Each mode offers different levels of control, investment, and risk, and companies typically choose a mode based on factors like the type of product, market conditions, regulatory environments, and the company’s resources.*”

- Class 1

This class emphasizes business strategies; goal alignment; and risk management. It is an important class for discussing business objectives and strategies in an international context.

It comprises 18,7% (f = 6 ST) of the total corpus analyzed. This class is composed of words such as “resource” (χ^2^ = 35,444); “target” (χ^2^ = 27,607); “goal” (χ^2^ = 27,551), and “strategy” (χ^2^ = 10.8).

Risk management is a fundamental component that ties directly into business strategy, helping companies achieve their objectives while maintaining a strong awareness of regulatory requirements. By integrating risk management into their strategies, businesses can proactively address potential challenges, ensuring growth and compliance in an increasingly complex global environment.

The participant understands that

“*When a company decides to internationalize and enter another country’s market, it must carefully consider various strategies to succeed in the foreign environment. These strategies are shaped by factors such as the nature of the business, target market characteristics, competition, regulatory requirements, and the company’s resources.*”

- Class 4

This class reflects daily challenges in complex contexts, such as “pharmaceutical regulations” and “regional dynamics”. It is a class that seems to deal with the diversity and complexity of the regulatory environment.

It comprises 16.7% (f = 6 ST) of the total corpus analyzed. This class is composed of words such as “big” (χ^2^ = 37.8); “challenge” (χ^2^ = 21.6); “regulatory” (χ^2^ = 5934); “pharmaceutical” (χ^2^ = 3506); and “complexity” (χ^2^ = 635)

The focus on pharmaceutical regulations and regional dynamics indicates a thorough exploration of how companies must manage legal, compliance, and operational hurdles while adapting to diverse environments.

The participant understands that

“*The biggest daily challenge in the pharmaceutical segment is balancing regulatory compliance with innovation and market demands, while ensuring product safety, quality, and timely delivery in a highly competitive and constantly evolving environment.*”

- Class 5

This class addresses issues related to “management,” “safety,” “research,” and “regulation.” The segments in this class seem to emphasize the need to maintain regulatory standards and requirements.

It comprises 16,7% (f = 6 ST) of the total corpus analyzed. This class is composed of words such as “ensure” (χ^2^ = 4056); “manage” (χ^2^ = 21.6); “safety” (χ^2^ = 15,913); “compliance” (χ^2^ = 2935); and “regulation” (χ^2^ = 1571).

The interconnections between management, safety, research, and regulation are critical pillars for ensuring a company’s success, particularly in highly regulated industries like pharmaceuticals, healthcare, or manufacturing. The emphasis on regulatory standards and requirements reflects the importance of maintaining compliance in an increasingly complex and globalized business environment.

The participants understand that

“*Regulatory requirements vary by country and can be particularly difficult to manage, as they involve multiple layers of compliance, including clinical trials, product approvals, manufacturing standards, labeling, and post-market surveillance.*”

“*The challenge is to stay up-to-date with evolving regulations, ensure full compliance across multiple jurisdictions, and manage the risk of non-compliance, which can lead to delays, fines, or product recalls. Additionally, ensuring the safety, efficacy, and quality of products while meeting regulatory demands adds another layer of complexity.*”

- Class 3

This class is associated with “international expansion”, “market” and “entry strategies”. The focus is on knowledge and strategies to operate in new markets and global contexts.

It comprises 14,8% (f = 6 ST) of the total corpus analyzed. This class is composed of words such as “knowledge” (χ^2^ = 16,921); “expand” (χ^2^ = 15,135); “foreign” (χ^2^ = 11,418); and “market” (χ^2^ = 2683).

Expanding into international markets comes with significant opportunities and challenges. The class likely aims to provide the knowledge and strategic tools businesses need to thrive in different regions and to adapt to local conditions while maintaining global objectives.

The participant understands that

“*Entering a foreign market requires a well-thought-out internationalization strategy that aligns with a company’s resources, goals, and target market conditions. There are several key strategies that businesses can adopt, each with its own advantages and challenges.*”

- Class 2

This is the smallest class in percentage. It covers “distribution”, “operating modes” and “pharmaceutical products”. It appears to be focused on logistics and the operational aspects of the sector.

It comprises 13% (f = 6 ST) of the total corpus analyzed. This class is composed of words such as “distribution” (χ^2^ = 21,948); “mode” (χ^2^ = 17,504); “regulatory” (χ^2^ = −17,465); “international” (χ^2^ = 16,085); and “operation” (χ^2^ = 5252)

It concerns logistics and operational aspects of the pharmaceutical industry, specifically dealing with distribution, operating modes, and pharmaceutical products. In an industry as complex and regulated as pharmaceuticals, efficient distribution and the right operational models are critical for ensuring that products reach consumers safely, on time, and in compliance with regulations.

The participant understands that

“*Ensuring products meet local and international regulatory standards, while also dealing with production, distribution, and market competition, can be a complex balancing act. Additionally, managing costs while maintaining product quality and safety is a critical ongoing issue.*”

### 3.2. Hierarchy and Relationship Between Classes

The hierarchy represented in the dendrogram shows how the classes were divided from a common trunk, reflecting the semantic proximity between them. Class 4 and Class 5 are closer together, indicating a thematic relationship between regulatory challenges and standards management. Class 2 and Class 3 also show proximity, suggesting that the themes of operations and international expansion are interconnected. And Class 1 and Class 6 share a focus on strategies and economic conditions, but with different approaches.

### 3.3. Word Cloud

**Figure 4:**
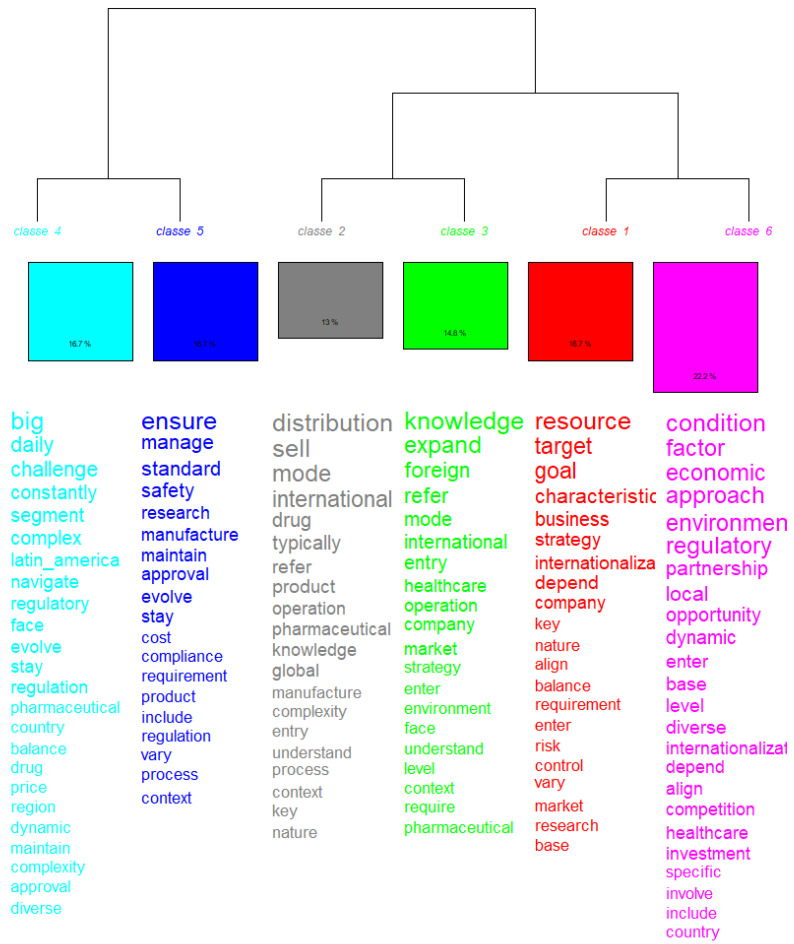
Descending hierarchical classification per word. Source: Researcher (2022).

The word cloud presented in Figure 4 is a visual representation of the most frequent words in the analyzed corpus. The most evoked words were “market” (f = 66), “pharmaceutical” (f = 50), “company” (f = 46), and “regulatory” (f = 35).

- Larger words (larger size):

“pharmaceutical”: Refers to the pharmaceutical sector, which is the central theme of the corpus. Indicates that the analysis is focused on issues related to this sector.

“market”: Highlights the importance of the market in the context of the corpus, possibly related to strategies for market entry, expansion or regulation.

“company”: Represents the relevance of companies and organizations in the context of the pharmaceutical sector and its operations.

“regulatory”: Emphasizes the importance of regulations in the sector, a recurring theme in analyses of this type.

“strategy”: Points to the discussion of business and regulatory strategies.

“international”: Indicates that the corpus addresses global issues, such as international expansion, regulations in different countries and foreign markets.

These larger words indicate the central themes of the corpus: pharmaceutical market, companies, regulation and international strategies.

- Medium-sized words:

“product,” “compliance,” “environment,” “country,” “healthcare,” “distribution”: These words suggest subtopics related to pharmaceutical operations, regulatory compliance, business environment, distribution, and healthcare.

“challenge,” “complexity,” “entry,” “risk”: These indicate challenges faced by the industry, such as regulatory challenges, market complexity, risks associated with entering new markets, and other critical factors.

These words complement the main themes, diminishing the practical aspects and challenges faced by companies in the pharmaceutical industry.

- Smaller words (lower frequency, but still significant):

“innovation,” “investment,” “align,” “latin_america,” “develop,” “nature”: These point to more specific topics, such as innovation, investment, product development, strategic alignment, and regional markets (such as Latin America). “price”, “quality”, “license”, “approval”: These are related to regulatory and economic issues, such as product approval, prices and quality.

### 3.4. Similitude Analysis

**Figure 5:**
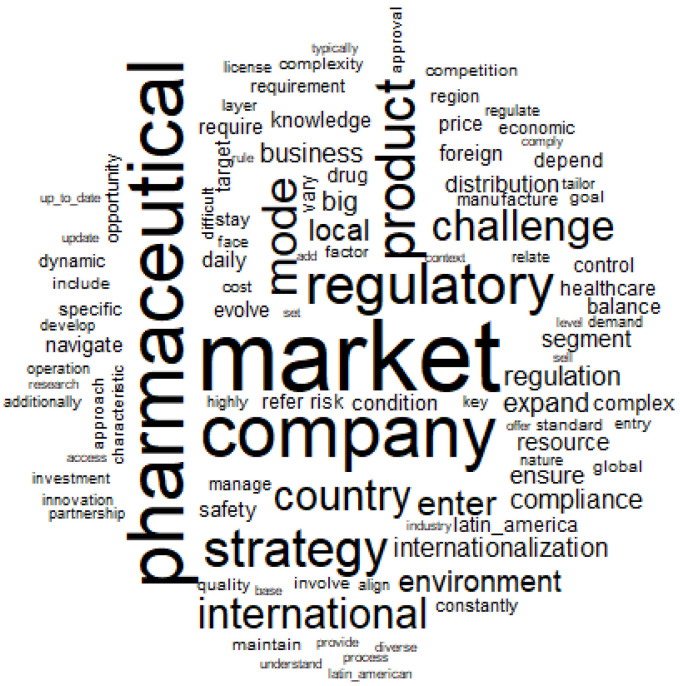
Word cloud. Source: Researcher (2022).

The similarity analysis presented in Figure 5 is a representation of the connections between the main terms in the corpus, based on the co-occurrence of words in the text segments. This analysis allows us to identify the semantic and thematic relationships between the terms, helping us to understand the central themes and the most relevant associations in the text (Figure 5).

- Central Word: “market”

The word “market” (mercado) is in the center of the graph, as it is the most relevant and connected term in the corpus. This means that the discussions in the text revolve around the market, which is the main axis of the analyses and relationships between the other terms.

The graph presents different branches connected to the central word, forming thematic subgroups.

Each of these groups represents a theme or aspect related to the market. Let’s break down the main groups.

The “Regulatory” subgroup (Regulatory) is associated with the words: “regulatory”, “challenge”, “compliance”, “requirement”, “approval”, “complex”, and “latin_america”. This group addresses regulatory issues and challenges faced by companies in the pharmaceutical market. Words such as “compliance”, “requirement” (requirements) and “approval” (approval) indicate the emphasis on legal and regulatory requirements, while “challenge” (challenges) and “complex” (complexity) highlight the associated difficulties. The relationship with “market” shows that regulations are an important factor in operating in the market, especially in specific regions such as Latin America (“latin_america”).

The subgroup “Pharmaceutical” (Pharmacist) is associated with the words: “pharmaceutical”, “innovation”, “opportunity”, “segment”, “drug”, and “industry”. This group focuses on the pharmaceutical sector, highlighting aspects such as innovation, market opportunities, specific segments and the role of drugs. The word “industry” (industry) reinforces the context of the sector analyzed. The relationship with “market” indicates that the pharmaceutical market is characterized by innovations and opportunities, but also by challenges related to segmentation and product development.

The “Product” subgroup (Product) is associated with the words: “product”, “quality”, “standard”, “safety”, “cost”, “manage”, and “maintain”. This group addresses issues related to pharmaceutical products, such as quality, standards, safety and costs. Terms such as “manage” (manage) and “maintain” (keep) suggest concern with the management of products in the market. The relationship with “market” reflects the importance of ensuring that products meet the standards required to compete effectively in the market.

The “International” subgroup (International) is associated with the words: “international”, “strategy”, “entry”, “distribution”, “partnership”, and “investment”. This group addresses internationalization strategies, including entry into new markets, distribution and partnerships. The word “investment” (investment) indicates the need for resources for global expansion. The relationship to “market” shows that international expansion is an essential part of market strategies, especially in the pharmaceutical sector.

The “Company” subgroup (Company) is associated with the words: “company”, “business”, “expand”, “resource”, “control”, and “location”. This group focuses on companies and their operations, including expansion, resource control and adaptation to local contexts. The relation to “market” highlights the role of companies in market dynamics and the need for adaptive strategies to operate in different regions.

The “Country” subgroup (Country) is associated with the words “country”, “economic_condition”, “risk”, “specific”, and “price”. This group addresses contextual factors, such as economic conditions, risks and prices, which vary by country. The relation to “market” indicates that country-specific economic conditions and risks influence market operations.

### 3.5. Key Points of Analysis

The word “market” connects all subgroups, underscoring that the market is the unifying theme of the corpus. The subgroups show that the corpus addresses a wide range of topics, including regulations, products, international strategies, business operations, and economic contexts. The mention of “latin_america” and “country” suggests that the corpus also explores regional and national specificities.

The Subgroup “Strategy” (Strategy) is associated with the words “entry”, “internationalization”, “key”, and “partnership”. This subgroup addresses strategic aspects related to the market, with an emphasis on:

- “Entry” (entry): Strategies for entering new or emerging markets.
- “Internationalization” (internationalization): Processes of global expansion, including adaptation to different markets.
- “Partnerships”: Strategic collaborations and alliances, essential to overcome barriers and seize opportunities.
- “Key”: Refers to critical or priority elements for strategic success.

The subgroup “strategy” is connected to the central word “market”, indicating that success in the market depends on well-planned strategies, especially in international and regulatory contexts.

### 3.6. Specificity and Correspondence Factor Analysis (CFA)

**Figure 6:**
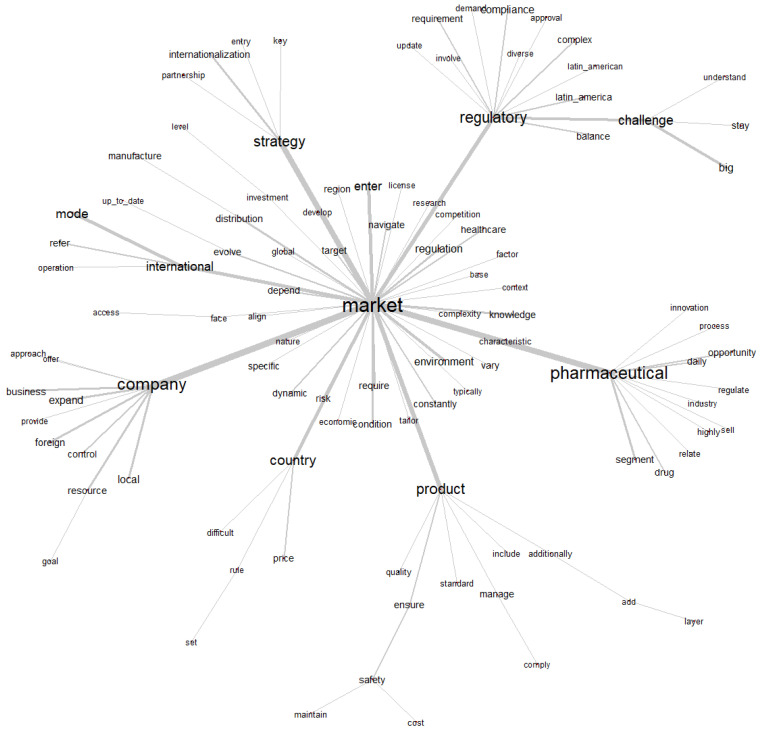
Spatial distribution of words and classes on a two-dimensional Cartesian plane. Source: Researcher (2022).

The CFA presented in Figure 6 shows the spatial distribution of words and classes on a two-dimensional Cartesian plane, allowing the visualization of the relationships between the different elements of the corpus.

**Figure 7:**
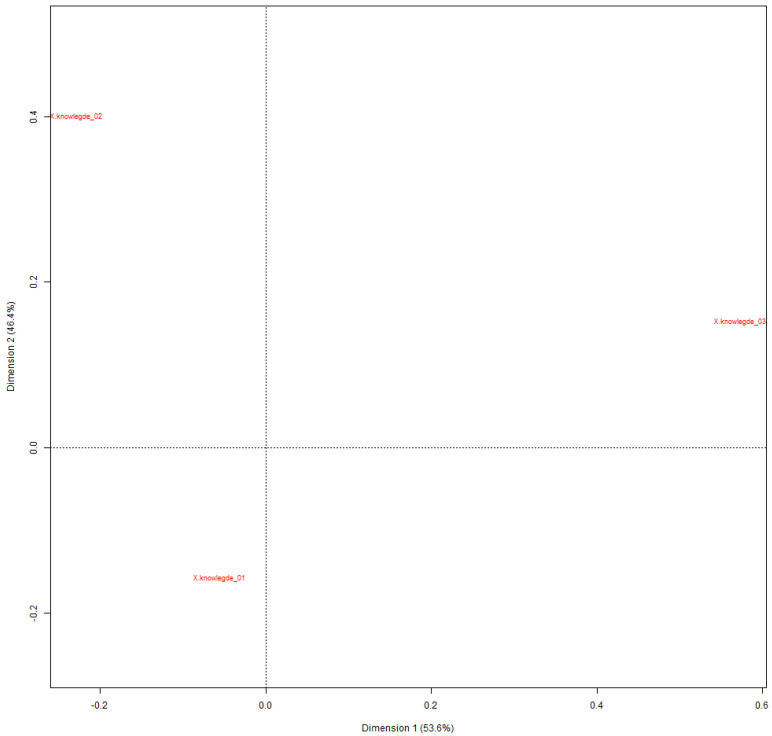
Dimension horizontal axis. Source: Researcher (2022).

The general characteristics of the graph (Figure 7) describe the dimensions, where Factor 1 (horizontal axis) explains 53.64% of the variance, Factor 2 (vertical axis) explains 46.36% of the variance, and the total variance explained is 100% (53.64% + 46.36%).

#### Word Distribution

**Figure 8:**
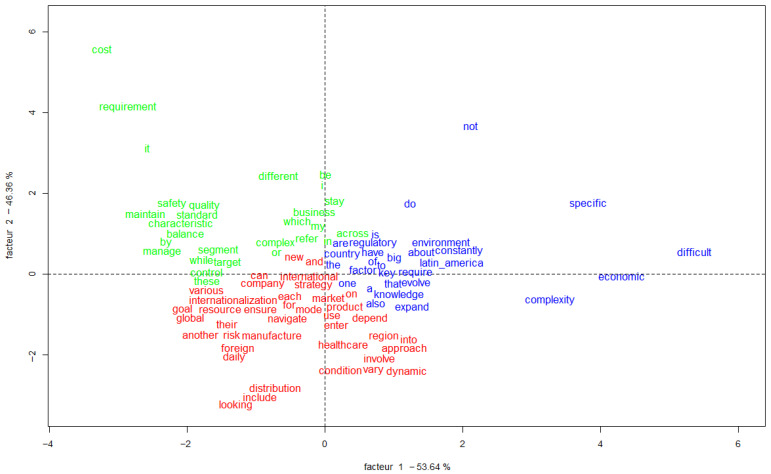
Dimension vertical axis. Source: Researcher (2022).

The graph (Figure 8) presents three groups of words, identified by different colors.

- Words in Green (Upper Left Quadrant):

Focus on operational and technical aspects.

Main terms: “cost”, “requirement”, “safety”, “quality”, “standard”.

Related to quality requirements and standards.

- Words in Red (Lower Left Quadrant):

Focus on strategy and internationalization.

Main terms: “strategy”, “internationalization”, “resource”, “distribution”.

Related to management aspects and global expansion.

- Words in Blue: management aspects and global expansion.
- Words in Blue (Upper Left Quadrant):

Focus on operational and technical aspects.

Focus on challenges and complexity.

Main terms: “specific”, “difficult”, “complexity”, “environment”.

Related to contextual aspects and challenges.

Quadrant Analysis

- Upper Right Quadrant:

Concentrates words related to specificities and challenges.

Strong presence of terms such as “specific”, “difficult”.

- Upper Left Quadrant:

Groups technical and operational aspects.

Emphasis on quality and requirements.

- Lower Left Quadrant:

Focus on strategy and international management.

Concentrates terms related to internationalization processes.

- Lower Right Quadrant:

Aspects related to market complexity and dynamics.

Terms that indicate change and adaptation.

### 3.7. Knowledge Points

The second graph shows the distribution of three knowledge points:

Knowledge_01 (understands the regulatory process): Located in the lower left quadrant.

Knowledge_02 (understands little about the regulatory process): Positioned in the upper quadrant left.

Knowledge_03 (does not understand the regulatory process): Located in the right quadrant.

### 3.8. Interpretation of Dimensions

- Dimension 1 (53.64%):

Horizontal axis in Figure 7.

Contrasts operational/strategic aspects (left) with challenges/complexities (right).

Main dimension of variation in the corpus.

- Dimension 2 (46.36%):

Vertical axis in Figure 8.

Contrasts technical/regulatory aspects (top) with strategic/international aspects (bottom).

## 4. Discussion

In this study, the knowledge contributions from stakeholders offered valuable insights, discoveries, and strategies relevant to the pharmaceutical sector. The preliminary questions used in the case study provided a robust foundation for understanding the crucial role stakeholders play in shaping regulatory practices, supporting internationalization strategies, and informing decision-making processes. Expanding the case study further will be essential to derive meaningful conclusions and to formulate informed recommendations that enhance knowledge-sharing, collaboration, and policy development within the pharmaceutical industry.

As highlighted by Hitt et al. [5], stakeholders are key actors in knowledge exchange regarding internationalization. Their contributions cover diverse market entry pathways—including exporting, licensing, alliances, and start-up modes—offering a holistic view of the globalization of the pharmaceutical industry. This knowledge is particularly valuable for policymakers, regulatory authorities, and industry leaders, as it supports the development of strategies and policies that foster growth, competitiveness, and sustainable innovation on a global scale.

It is also important to emphasize the stakeholders’ perceptions regarding regulatory harmonization, which align with the findings of Aggarwal [12], who underscore the role of regional convergence in facilitating market access. Our results reinforce this perspective, while adding novel evidence on how fragmentation across Latin American regulatory frameworks continues to create barriers. Furthermore, the findings related to strategic adaptation resonate with the discussions of Nonaka et. al [3], but also extend the literature by revealing the significance of informal networks, local partnerships, and relational trust—elements that remain underexplored in current research on regulatory internationalization.

The present study contributes to the literature by examining knowledge management strategies in the context of regulatory processes and internationalization in the pharmaceutical sector. By combining lexical and content analysis with qualitative interviews, we were able to uncover patterns of stakeholder knowledge-sharing that illuminate both the opportunities and challenges of regulatory harmonization.

Through in-depth interviews with stakeholders from multiple Latin American countries, the study captured a broad spectrum of perspectives on regulatory knowledge, internationalization strategies, harmonization efforts, and industry challenges. This comprehensive approach not only highlights convergences with existing studies but also identifies gaps and novel insights, thereby advancing understanding of the complex interplay between regulatory frameworks, stakeholder knowledge, and global pharmaceutical expansion.

Our study delved deeply into each stakeholder group’s thoughts and viewpoints through interviews. This comprehensive approach enabled us to understand perspectives on regulatory knowledge, internationalization strategies, harmonization efforts, and industry challenges.

## 5. Limitations and Future Work

This study has some limitations that should be acknowledged. First, although the sample included diverse stakeholders from multiple Latin American countries, the number of participants was limited, and therefore the findings cannot be generalized across the entire region. Language differences and potential interpretation biases may also have influenced the depth of some responses. In addition, while the use of IRaMuTeQ software strengthened the lexical and content analysis, its technical focus may have restricted the exploration of more nuanced narratives.

Future research should expand the number and diversity of participants, incorporating additional perspectives from regulators, industry representatives, and policymakers across different regions. Comparative studies between Latin America, the European Union, and other emerging markets would also provide valuable insights into how harmonization, health technology assessment (HTA), and reimbursement policies impact pharmaceutical internationalization. Finally, further investigation into the role of informal networks, partnerships, and local knowledge strategies could deepen understanding of how stakeholders contribute to shaping regulatory and market-access strategies globally.

## 6. Conclusions

The results of this study underscore the importance of regulatory knowledge for professionals’ support and implementation of international strategies. Regulatory knowledge provides professionals with tools and insights to navigate complex regulatory environments, make informed decisions, and enhance organizational performance in global markets.

The importance of local knowledge stems from the understanding that markets differ across countries due to cultural, economic, legal, and social factors. Gaining deep insights into these nuances allows companies to adapt products, marketing strategies, and business practices to meet the specific needs and preferences of the local market. Tacit knowledge acquired through experience and interaction with the local environment provides a competitive advantage in navigating these complexities effectively.

## Figures and Tables

**Table 1:** Regulatory agencies known for Latin America.

Countries	Health Authorities	Regulatory Differences
Latin American		
Argentina	Administración Nacional de Medicamentos, Alimentos y Tecnología (ANMAT)	
Bolivia	Unidad de Medicamentos y Tecnología en Salud (UNIMED)	Decentralized regulatory requirements. Each country has a specific health framework.
Brazil	Agencia Nacional de Vigilancia Sanitaria (ANVISA)	
Chile	Agencia Nacional de Medicamentos (ANAMED)	
Colombia	Instituto Nacional de Vigilancia de Medicamentos y Alimentos (INVIMA)	
Costa Rica	Ministerio de Salud (MNSA)	
Cuba	Centro para el control estatal de medicamentos y dispositivos médicos (CECMED)	
Dominican Republic	Dirección General de Drogas y Farmacia	
Ecuador	Agencia Nacional de Regulación, Control y Vigilancia Sanitaria (ARCSA)	
El Salvador	Dirección Nacional de Medicamentos (DNM)	
Guatemala	Departamento de Regulación y Control de Productos Farmacéuticos y Afines	
Honduras	Agencia de Regulación Sanitaria (ARSA)	
Mexico	Comisión Federal para la Protección contra Riesgos Sanitarios (COFEPRIS)	
Nicaragua	Dirección General de Regulación Sanitaria	
Panama	Dirección Nacional de Farmacias y Drogas (DNFD)	
Paraguay	Dirección Nacional de Vigilancia Sanitaria	
Peru	Dirección General de Medicamentos Insumos y Drogas (DIGEMID)	Decentralized regulatory requirements. Each country has a specific health framework.
Puerto Rico	Departamento de Salud	
Haiti	Ministerio de Salud	
Uruguay	Ministerio de Salud Pública (MSP)	
Venezuela	INHRR: Instituto Nacional de Higiene Rafael Rangel (INHRR)	

Source: Researcher (2022).

**Table 2:** Regulatory knowledge for successful internationalization.

**Internationalization knowledge**	*“Large/multinational companies with capacity for growth and expansion […]”*
	*“Easier to entry the market […]”*
	*“The companies who have the regulatory knowledge are more compliant to rules and regulations of another country […]”*
	*“By learning more regulations, products can now be formulated to meet various regulations […]”*
	*“Regulatory knowledge is important to entry in any country […]”*

Source: Research data (2022).

## Data Availability

The original contributions presented in this study are included in the article. Further inquiries can be directed to the corresponding authors.
